# Epidemiology of Tuberculosis in Multi-Host Wildlife Systems: Implications for Black (*Diceros bicornis*) and White (*Ceratotherium simum*) Rhinoceros

**DOI:** 10.3389/fvets.2020.580476

**Published:** 2020-11-04

**Authors:** Rebecca A. Dwyer, Carmel Witte, Peter Buss, Wynand J. Goosen, Michele Miller

**Affiliations:** ^1^Division of Molecular Biology and Human Genetics, Faculty of Medicine and Health Sciences, Department of Science and Innovation - National Research Foundation Centre of Excellence for Biomedical Tuberculosis Research, South African Medical Research Council Centre for Tuberculosis Research, Stellenbosch University, Cape Town, South Africa; ^2^Disease Investigations, San Diego Zoo Global, San Diego, CA, United States; ^3^Veterinary Wildlife Services, Kruger National Park, Skukuza, South Africa

**Keywords:** epidemiology, *Mycobacterium bovis*, rhinoceros, TB risk, TB transmission, tuberculosis

## Abstract

Cases of tuberculosis (TB) resulting from infection with *Mycobacterium tuberculosis* complex (MTBC) have been recorded in captive white (*Ceratotherium simum*) and black (*Diceros bicornis*) rhinoceros. More recently, cases have been documented in free-ranging populations of both species in bovine tuberculosis (bTB) endemic areas of South Africa. There is limited information on risk factors and transmission patterns for MTBC infections in African rhinoceros, however, extrapolation from literature on MTBC infections in other species and multi-host systems provides a foundation for understanding TB epidemiology in rhinoceros species. Current diagnostic tests include blood-based immunoassays but distinguishing between subclinical and active infections remains challenging due to the lack of diagnostic techniques. In other species, demographic risk factors for MTBC infection include sex and age, where males and adults are generally at higher risk than females and younger individuals. Limited available historical information reflects similar age- and sex-associated patterns for TB in captive black and white rhinoceros, with more reports of MTBC-associated disease in black rhinoceros than in white rhinoceros. The degree of MTBC exposure in susceptible wildlife depends on their level of interaction, either directly with other infected individuals or indirectly through MTBC contaminated environments, which is dependent on the presence and abundance of infected reservoir hosts and the amount of MTBC shed in their excreta. Captive African rhinoceros have shown evidence of MTBC shedding, and although infection levels are low in free-ranging rhinoceros, there is a risk for intraspecies transmission. Free-ranging rhinoceros in bTB endemic areas may be exposed to MTBC from other infected host species, such as the African buffalo (*Syncerus caffer*) and greater kudu (*Tragelaphus strepsiceros*), through shared environmental niches, and resource co-utilization. This review describes current knowledge and information gaps regarding the epidemiology of TB in African rhinoceros.

## Introduction

Tuberculosis (TB) is a chronic infectious disease that affects a broad range of host species (1). It is caused by members of a group of closely related pathogenic mycobacteria known as the *Mycobacterium tuberculosis* complex (MTBC) (1). Bovine tuberculosis (bTB) is caused by *Mycobacterium bovis* (*M. bovis*), which is known to infect livestock as well as captive and free-ranging wildlife species (2, 3). For most wildlife, however, little is known about susceptibility to MTBC infection, pathogenesis, and its impact on affected populations.

Rhinoceros are iconic species which are under threat due to habitat destruction and heavy poaching pressure. Cases of TB resulting from infection with *M. tuberculosis* and *M. bovis* have been recorded in captive, semi-captive (maintained in private reserves and more intensively managed), and free-ranging rhinoceros worldwide (4–19); TB was implicated as the cause of death in some of these cases.

Kruger National Park (KNP) and Hluhluwe–iMfolozi Park (HiP) are home to large populations of free-ranging black (*Diceros bicornis*) and white (*Ceratotherium simum*) rhinoceros in South Africa. As of 2016, HiP housed ~1,500 white rhinoceros and 360 black rhinoceros (20). In 2017, the Kruger National Park contained ~5,150 white rhinoceros and 500 black rhinoceros (21), at which time the global populations totaled 20,300 white rhinoceros and 5,200 black rhinoceros (22). The HiP and KNP populations have been and continue to be central to the “Integrated Strategic Management of Rhinoceros” plan, introduced by the South African Department of Environmental Affairs (23, 24). Part of this strategy relies on the translocation of rhinoceros from the feeder populations in these parks to newly developing rhinoceros safeguarding strongholds around the country. However, KNP and HiP are endemic for bTB, and these rhinoceros populations share habitat ranges and various resources with *M. bovis–*infected wildlife (over 20 species in KNP), including important bTB maintenance hosts such as African buffaloes (*Syncerus caffer*) (2), and greater kudu (*Tragelaphus strepsiceros*) (25–27). The identification of disease in free-ranging wildlife is often challenging due to limited resources and access to these populations for diagnostic testing, and it was only with the increase in poaching and associated veterinary interventions that evidence of MTBC infection in white and black rhinoceros in KNP was discovered (9, 19).

Although *M. bovis* and *M. tuberculosis* have not been considered an immediate threat to the world's African rhinoceros populations, the potential impact of these pathogens on their health and conservation is largely unknown. Because bTB is a World Organization for Animal Health (OIE) and nationally notifiable disease (1), animals with *M. bovis* infection are subject to regulatory requirements which limit their movements between populations; this hampers conservation efforts that are reliant on translocation of rhinoceros from bTB-endemic to bTB-free areas.

Since the discovery of *M. bovis* infection in the free-ranging rhinoceros populations in KNP (13, 23), knowledge gaps regarding the risk of MTBC infection, intra- and inter-species transmission, and disease progression in these species have become apparent. This review describes the current knowledge regarding TB in African rhinoceros and provides information on epidemiological aspects of this disease in other relevant species, especially free-ranging populations, to improve understanding of the disease and inform management strategies.

## Assessment of Infection Risk in African Rhinoceros

With any infectious disease, the risk of becoming infected is based on an individual's susceptibility when exposed to an infectious dose of the etiological agent, as well as the likelihood of exposure to the pathogen (28). These factors provide a foundation for investigating infection risks and will be discussed as they pertain to *M. tuberculosis* and *M. bovis* infection in black and white rhinoceros.

### Outcomes and Detection of MTBC Infections: Epidemiological Implications

MTBC infections are typically chronic and once disease occurs, usually progressive. In various human and animal hosts, infections have been observed to move between various stages in a dynamic host-pathogen interaction network, where clinical manifestations of the infection vary between stages (29–32). Following infection, the host's innate immune system may eliminate the mycobacteria. If the mycobacteria are not eliminated, T helper type-1 cell-mediated immune (CMI) responses develop and are followed by the T helper type-2 humoral immune response through B-lymphocyte activation and an increase in circulating antibodies (33–35). Immunological responses of the host play a key role in determining the outcome of infection, and in humans, these include latent, incipient and subclinical stages (30). These stages have not been clearly defined in animal hosts, but subclinical MTBC infections have been reported in a variety of species (36–39). Although controversial, there is speculation that latent infection of animals with *M. bovis* may also occur (35, 40). These complex and dynamic interactions between host and pathogen can lead to elimination of infection, or an asymptomatic stage in which the mycobacteria are either dormant or result in localized disease, or progression to active disease, which has been observed in a multitude of species (29, 30, 41, 42).

**Figure 1:**
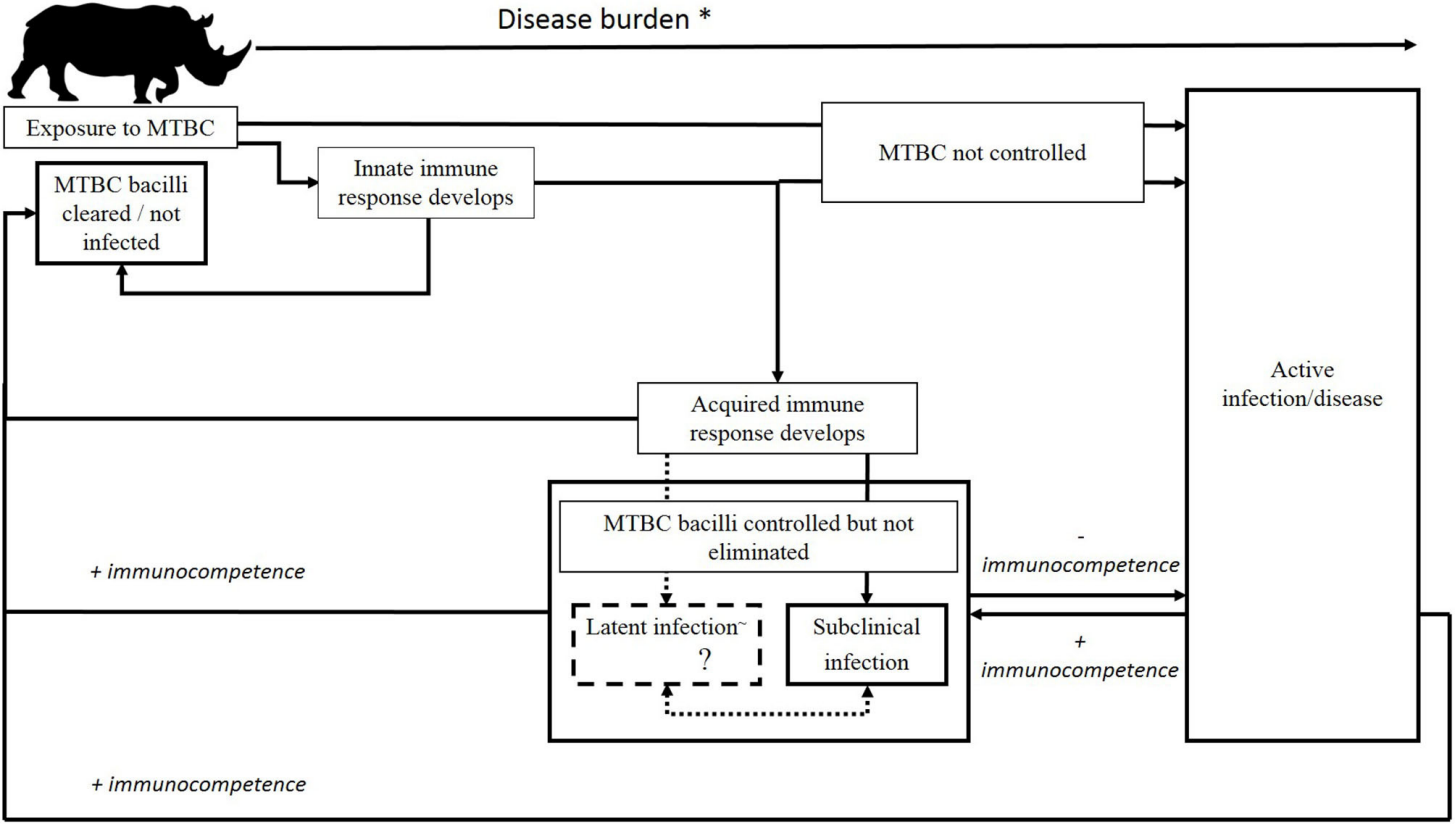
Possible outcomes of *M. bovis* infection in African rhinoceros. After initial exposure, MTBC may be eliminated by the host's immune response, persist as a subclinical or latent^~^ infection, or progress to active infection/disease. Following the establishment of subclinical or latent infection, the host's immune response may clear the MTBC, or infection may persist in this form, either naturally progressing in a slow or rapid fashion to active tuberculosis, or cycling through subclinical and latent states, before development into symptomatic disease or eventual eradication of the infection by the host's adaptive immune response. ^*^Rising disease burden implies an increase in abundance of TB and/or MTBC biomarkers, immunological changes characteristic of stage of infection, and increasing pathology, with a declining recovery prognosis. ^~^Although controversial, there is speculation that latent infection of animals with *M. bovis* may occur. More research is required.

Although there is a paucity of information on outcomes of MTBC infection in rhinoceros, a hypothesized scenario is shown in Figure 1; however, further investigation is required to verify these stages. Most cases of TB in zoos have only been detected once disease is sufficiently advanced to detect clinical signs, resulting in the death of the rhinoceros either due to euthanasia or disease complications (Supplementary Table 1) (4–6, 17). However, a study involving three experimentally *M. bovis*-infected white rhinoceros, monitored serially over 2 years, suggests that although immunological responses could be detected, the animals appeared to contain, and potentially eliminate, the infection (43, 44). Similarly, in the limited cases of natural *M. bovis* infection in white rhinoceros, pathological lesions were localized in lymph nodes or other tissues (19). The outcome of infection appears to be more complex than simply a progression to disease, based on these observations as well as reports of immunological responses (without evidence of disease) in rhinoceros that have been exposed to other known TB cases (7, 13, 15). In addition, decreases in immunocompetence as a result of comorbidities, drought, capture/transport–induced stress, increased age, or other factors may be associated with greater susceptibility to disease as sequelae of acute infection or activation of subclinical infection in rhinoceros, although tools to identify these stages need to be developed.

In order to characterize the epidemiology of TB in a population, it is important to have accurate diagnostic methods that can distinguish between various stages of infection and disease, since animals in different stages of infection may present with altered levels of transmission risk (45). Risk factors associated with acquiring an infection may be different from those that increase the likelihood of disease progression, or the maintenance of a subclinical or latent infection (28, 30). This important distinction may have implications for understanding the epidemiology of TB and could impact subsequent management decisions.

*In vivo* and *in vitro* indirect detection methods for early MTBC infection primarily rely on the detection of TB-specific adaptive immune responses of the host, including the tuberculin skin test (TST) and MTBC antigen stimulated cytokine assays (46, 47). In addition, serological assays for the detection of host-specific antibodies to MTBC antigens have been useful for diagnosis of TB in certain animal species (48–50), although they are considered unreliable for TB diagnosis in humans (51). In rhinoceros, the TST is unreliable due to cross-reactivity with environmental mycobacteria (7, 13, 52); therefore, a white rhinoceros whole blood MTBC antigen-specific interferon-gamma release assay (IGRA) for *M. bovis* infection has recently been developed (44, 53). Serological assays for the detection of antigen-specific antibodies have also been shown to be useful for the diagnosis of MTBC infection in rhinoceros (15, 18). However, the use of these indirect immunological diagnostic assays alone may not distinguish between recent infection, latent/incipient/subclinical infection, and active disease.

One way to overcome the challenges posed by these indirect tests is to directly detect the pathogen by mycobacterial culture and nucleic acid amplification tests (NAATs) (54, 55). Mycobacterial culture and speciation are useful as both pre- and post-mortem diagnostic tests for MTBC infection. Ante-mortem samples obtained for culture include bronchoalveolar, tracheal and gastric lavages, as well as nasal and fecal swabs, although culture of tissue obtained during necropsy may be more sensitive for detection of bacilli. Although this method is highly specific, culture of ante-mortem samples has low sensitivity, which may be related to the site of infection and whether the individual is shedding at the time of sampling (56). For example, in a study evaluating shedding in three experimentally *M. bovis*-infected rhinoceros, only one of 36 tracheal lavage samples collected monthly over a 2-year period was *M. bovis* culture positive (43). In humans and recently in wildlife, mycobacterial culture has been supplemented with NAATs including the automated GeneXpert MTB/RIF Ultra qPCR assay (Ultra) (57, 58). This rapid ancillary test may enable the direct detection of MTBC DNA in some tissues (59), as well as animal respiratory samples (57). Regardless, the direct detection of MTBC organisms alone also does not provide information on the host's stage of infection or disease. The presence and classification of lesions detected by macroscopic and microscopic examination provides important information for staging disease, although this is primarily used post-mortem (43, 60).

Molecular methods such as spoligotyping, mycobacterial interspersed repetitive unit-variable number of tandem repeat (MIRU-VNTR) genotyping, or whole genome sequencing of *M. bovis* isolates may be useful not just for diagnosis, but also for tracing the origin of MTBC infection in multi-host systems (26, 61–64). The application of these techniques in rhinoceros and other free-ranging wildlife, though useful, is challenging due to limited samples. Nonetheless, these techniques have been employed to investigate the distribution and transmission of MTBC strains in some wildlife multi-host systems (63), including between brushtail-possums (*Trichosurus volpecula*) (65), badgers (65, 66), deer (67) or African buffaloes (68) and cattle at the livestock/wildlife interface (65–68). Therefore, use of both direct and indirect detection methods should be included in investigations of transmission in rhinoceros.

### Investigating Susceptibility of Black and White Rhinoceros to Infection With *M. tuberculosis* and *M. bovis*

**Figure 2:**
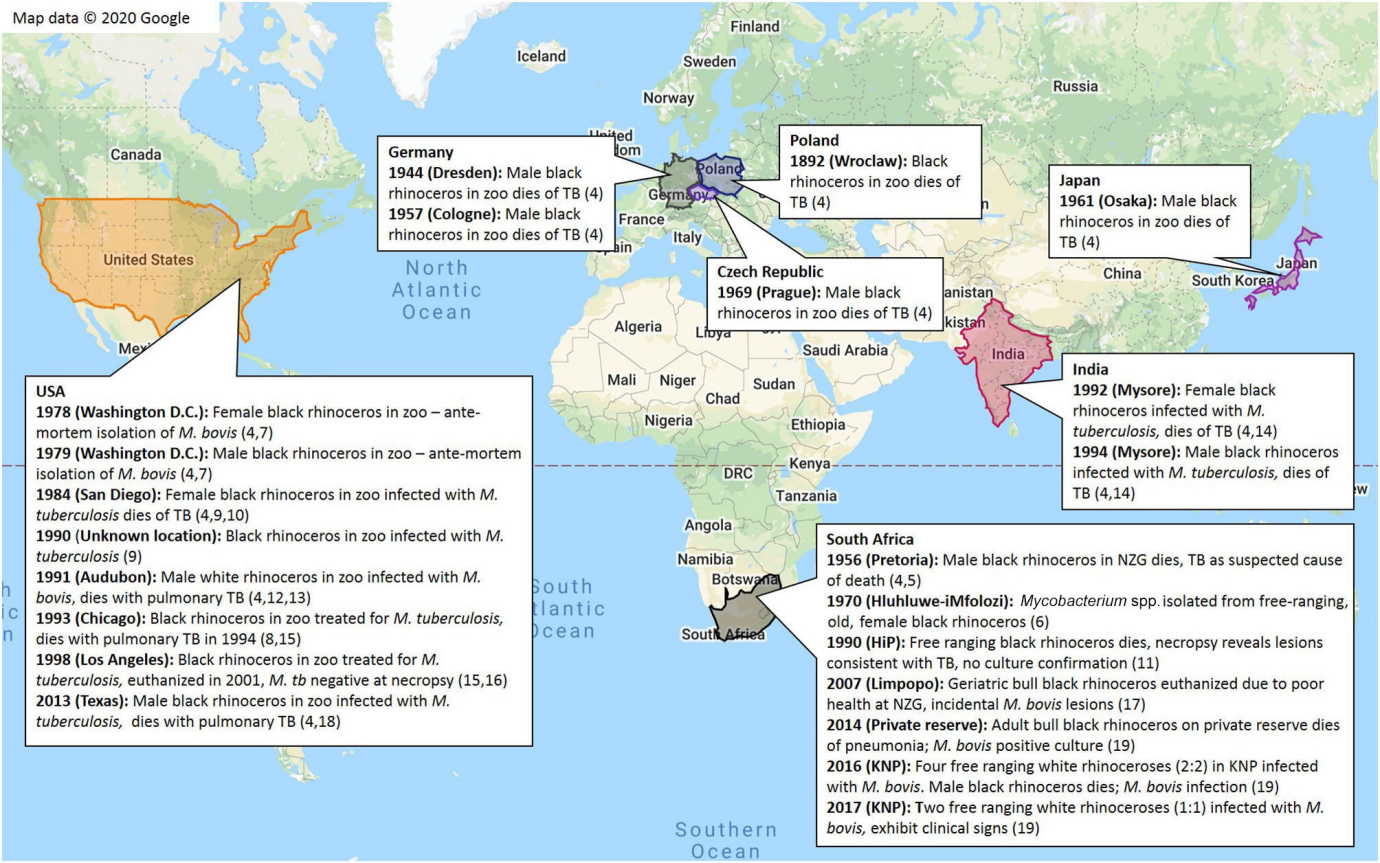
Summary of globally recorded historical cases of tuberculosis in black rhinoceros (*D. bicornis*) and white rhinoceros (*C. simum*) (4–19)*. *This data was located in the Rhinoceros Resource Center (RRC) literature database, or through extensive web searches with (9) as a guide.

As a result of the popularity of rhinoceros for zoological exhibition, they have historically been globally distributed through importation. Reports of TB in captive rhinoceros in zoological gardens worldwide date back to the late 1800's. Historical cases of bTB and TB in black and white rhinoceros are summarized in Figure 2. While TB is still considered a rare occurrence in domestic perissodactyls (69), these cases provide evidence for susceptibility of black and white rhinoceros.

According to limited information, most TB cases have been recorded in black rhinoceros, with an apparent paucity of cases identified in white rhinoceros (4–19). This observation may be related to differences in numbers, demographics, housing or management of these species in zoological collections, exposure to other infected animals, employees or visitors, differences in species-specific susceptibility to the different pathogens, or the impact of individual or species-specific co-morbidities on immunocompetence and TB susceptibility. Black rhinoceros in captivity are known to suffer from a variety of syndromes (70); afflicted individuals may have compromised immunity that increases their susceptibility to TB, which could explain the apparent higher prevalence in this species compared to white rhinoceros.

Most reports of TB in captive rhinoceros have been caused by infection with *M. tuberculosis*, with only a few caused by *M. bovis*. This is hypothesized to be a result of a high level of exposure of captive animals to *M. tuberculosis* through direct or indirect interactions with humans (especially in high TB burden countries), while in captivity or during exportation (4) (Supplementary Table 1). Another explanation might be differences in virulence of *M. tuberculosis* and *M. bovis* in rhinoceros, although limited studies suggest that *M. bovis* has greater virulence in other species, such as mice and goats, compared to *M. tuberculosis* (71, 72).

The susceptibility of white rhinoceros to *M. bovis* was studied in experimentally infected animals (43, 44). The results confirmed susceptibility to infection and potential to shed bacilli, albeit based on the detection of viable *M. bovis* in only one of 36 tracheal lavage samples collected over the course of the study. None of the individuals developed clinical signs or evidence of disease based on gross and histological examination, although *M. bovis* DNA was detected by PCR in lung tissues of two animals at necropsy (43). The immune response kinetics and pathological findings suggested that the rhinoceros were able to contain and possibly clear *M. bovis* infection (44). This observation is consistent with the historical lack of TB cases in white rhinoceros, which may be due to the ability to contain and clear the infection before the onset of disease. However, it should be noted that the response of rhinoceros to experimental inoculation in this study may not reflect naturally occurring infection, which could occur through one or more exposure events over time, each with variable numbers of MTBC bacilli. Additionally, the individuals in this study were not subject to stressful conditions and were young (4–7 years old); all individuals had adapted to living in managed care by the time of initial infection, and were not exposed to the seasonal variations in food availability that they might have been if free-ranging. It is unknown whether the lack of disease development in these white rhinoceros was a consequence of low susceptibility to disease resulting from *M. bovis* infection, or the conditions associated with the experimental infection. In contrast, a naturally *M. bovis* infected 29-year-old white rhinoceros in a zoo developed weight loss, cough and nasal discharge and succumbed to the infection (13), demonstrating that this species can develop disease.

In contrast to white rhinoceros, evidence of TB disease in black rhinoceros has been reported more frequently. An elderly (estimated 35–40 years old) black rhinoceros, euthanized due to loss of condition, had small non-encapsulated pulmonary granulomas associated with *M. bovis* infection (17). Similarly, the free-ranging black rhinoceros in KNP, infected with *M. bovis*, had evidence of significant pulmonary pathological changes (9). Interestingly, the first case was in an elderly animal and the second rhinoceros case was discovered after a prolonged period of drought. However, cases of *M. bovis*-associated disease in zoo black rhinoceros have been reported in animals that were ~20–25 years old (7). In addition, post-mortem pulmonary changes consistent with *M. tuberculosis* disease were observed in multiple zoo black rhinoceros aged 13–33 years old (14, 15, 18). The reports of clinical signs and presence of changes post-mortem in black rhinoceros infected with *M. bovis* or *M. tuberculosis* suggests this species may be more prone to TB disease.

### Assessing Risk Factors for Infection and Transmission Patterns of MTBC in African Rhinoceros Populations

#### Demographic Risk Factors

There is limited literature that characterizes risk factors of MTBC exposure and transmission patterns in free-ranging black and white rhinoceros populations, likely a result of the logistical and technical difficulties associated with disease surveillance and sporadic cases in these animals. Therefore, extrapolation from literature on TB in other species and multi-host systems may aid in understanding the epidemiology of MTBC in African rhinoceros populations. Demographic risk factors for MTBC infection, such as sex and age, have been described in humans, mice, cattle, and limited species of wildlife (48, 49, 73–82). Results from these studies may inform hypotheses regarding demographic patterns of MTBC infection in rhinoceros.

In many TB-susceptible species, sex is considered a risk factor for infection (48, 49, 73–78). In humans, the global male to female ratio for individuals that develop TB is 2:1 (73). While this has partially been due to socioeconomic and cultural barriers in access to healthcare (74), inherent biological characteristics are also implicated. Sex-based differences in susceptibility are usually observed in adults, but not in children or adolescents (83). This suggests that the relative male: female difference in susceptibility is related to the effect of steroid sex hormones and their regulatory activities on immune cells (84).

Both testosterone and progesterone are immunosuppressive. These hormones impair macrophage activation and may increase TB susceptibility (85, 86). In contrast, estrogen is a pro-inflammatory mediator that stimulates the production of tumor necrosis factor alpha (TNF-α) (87), and interacts with the IFN-γ promoter (88). In mice, increased susceptibility to TB has been observed in post-adolescent males relative to post-adolescent females, with this difference partially mitigated by castration (84).

In various wildlife species, studies have shown a higher frequency of MTBC infection in males, which could be linked to hormonal differences, but behavioral differences may also play a role. In a cohort of free-ranging African elephants (*Loxodonta africana*) tested in KNP, overall TB seroprevalence was higher in males than in females (48). Another study reported a higher risk for both bTB infection and disease in male badgers (*Meles meles*) than in females (75). An epidemiological study of white-tailed deer (*Odocoileus virginianus*) in Michigan also reported a higher odds of being bTB test-positive in males compared to females (76). This study also reported a dramatic effect of sex on the association of increasing age with positive TB test status. In fawns and yearlings, no significant difference in TB incidence between males and females was found; however, in age groups of 2 years and above, males were increasingly more likely to test positive for TB than females of the same age class. This is a similar trend to what is observed in humans, and may be due either to sex-based hormonal differences, or to the contrasting social/reproductive behavior between mature males and females (76, 89). Therefore, it may be difficult to separate risk factors associated with hormonal and behavioral differences in adults.

Most wildlife epidemiological studies report a higher risk of bTB in males than in females; however, this association does not strictly hold true for all species. Studies of different populations of wild boar, for example, have yielded conflicting results with respect to the association between sex and bTB risk. One study on wild boars in Portugal (82) reported a significantly higher bTB incidence in female than males in all age groups. In a different wild boar population in Spain (81), studies showed a significantly higher bTB prevalence in males. Studies of wild boar populations in France (77) and Italy (78) reported no significant association between sex and TB risk. Variable findings from different populations of the same species illustrate the complexity of determining sex-associated bTB risk. Hormone-derived TB susceptibility of a species may be largely conserved between different multi-host systems; however, sex may also be a mediator of pathogen exposure due to sex-related differences in social, reproductive, and territorial behavior as well as movement patterns. The degree to which such sex-related factors alter rhinoceros' exposure to MTBC may depend on the unique characteristics of dispersal and transmission in that particular host system (89).

Most historical reports of TB in captive rhinoceros have occurred in black rhinoceros males (Figure 2 and Supplementary Table 1). It is unknown whether this observation represents a true species and sex predilection for TB, or whether reports are biased for other reasons, such as the skewed natal sex ratios in captive black rhinoceros (90), or a disproportionate number of black rhinoceros and/ or males kept in zoos. However, available records show a preference for import and exhibition of female white rhinoceros over males, and no substantial preferential import of male vs. female black rhinoceros for exhibition during the twentieth century (4). Records for USA, Mexico, and Australia show a near-equivalent sex ratio of black rhinoceros currently kept in captivity (91, 92), and the sex ratio of white rhinoceros in captivity in Canada, the USA, Mexico, Chile, and Singapore is substantially skewed toward females (93). These records also indicate that a higher number of white rhinoceros are kept in captivity compared to black rhinoceros; there are currently 278 white rhinoceros in captivity in Canada, the USA, Mexico, Chile, and Singapore (93), compared to 96 black rhinoceros in the USA, Mexico, and Australia (91, 92). These data do not support an apparent bias toward males or black rhinoceros in captivity, which suggests that reports (Figure 2 and Supplementary Table 1) may reflect a true increased risk for TB in these groups. While the absolute historical numbers of rhinoceros housed in captivity globally are unknown, and therefore cannot be used to draw conclusions on TB risk in rhinoceros, these observations provide avenues for further investigation into species and sex-specific susceptibility.

In humans, TB occurs in individuals of all ages, although the highest burden is in men past adolescence (≥15 years old) (73). Human susceptibility to TB shows an increase with age, which may be due to age-related effects on the immune system or possibly the outcome of multiple exposures over time (79). A similar age-related bTB trend has been observed in cattle, with a peak in incidence after 12 months of age (80). Adult warthogs (*Phacochoerus africanus*) and African elephants (>25 years old) in bTB endemic regions also showed a higher seroprevalence than their younger counterparts (48, 49). Increasing age had the greatest effect on TB disease risk in a meerkat population in South Africa (94).

While most studies in wildlife show increasing bTB prevalence with age, studies conducted in the Iberian Peninsula report higher prevalence in juvenile wild boar than adults in high-prevalence multi-host systems (81, 95). This could be due to higher susceptibility in juveniles compared to fully-grown adults in this species, possibly related to immunological maturity, or age-related changes in behavior that result in increased exposure to the pathogen. Interestingly, historically reported cases of TB in captive African rhinoceros appear to have occurred exclusively in adults (Figure 2 and Supplementary Table 1). These observations suggest that infection can take years to manifest in these species (18), or that there is an increase in susceptibility and/or repeat exposures with age. Based on other species, it is likely that both sex and age are risk factors for MTBC exposure and infection in black and white rhinoceros.

#### Transmission of *M. bovis* in Multi-Host Systems

Investigation of TB transmission has been limited in free-ranging rhinoceros until recently because of a lack of diagnostic assays and paucity of samples. Therefore, characterization of transmission depends largely on extrapolation using patterns observed in other multi-host systems. Some of the predictors for persistence and transmission of a pathogen within a multi-host system appear to be related to patterns of movement, migration, and different modes of interactions between host species (28, 96, 97). Importantly, the presence of an infected reservoir species in the system has been shown to increase the risk of spill-over to other susceptible hosts. Wildlife reservoir hosts for bTB are present globally, including African buffaloes in South Africa (27) [and possibly other areas in Africa where the species occurs (98, 99)], greater kudu in South Africa (27), brush-tailed possums (*T. volpecula*) in New Zealand (100), European wild boar (*Sus scrofa*), red deer and fallow deer (*Dama dama*) in Spain (95), white-tailed deer (*O. virginianus*) in the USA (101), elk (*Cervus canadensis*) (102) and American bison (*Bison bison*) (103) in Canada, and European badgers (*M. meles*) in the United Kingdom (97). In wildlife populations with *M. bovis*, there are numerous examples of intra- and inter-species transmission (27, 104–107). Direct intra-species *M. bovis* transmission can occur through respiratory droplets in social species like African buffaloes (107) or through antagonistic or territorial behaviors like those that occur between white-tailed deer (76).

The mechanism of inter-species *M. bovis* transmission to herbivores is largely unknown but has been attributed to indirect interactions through contamination of pastures, feed, or browse with MTBC shed by infected hosts. Various studies have demonstrated that infected hosts shed *M. bovis* into the environment (106, 108). In one study, intranasal administration of *M. bovis* to calves resulted in intermittent shedding for up to 38 weeks (108). A European study of infected wild boar and red deer demonstrated shedding by oronasal, bronchial-alveolar, fecal and urinary routes (106). In that study, 83% of wild ungulates with bTB had mycobacteria isolated in at least one type of excretion, which suggests a high level of shedding into the environment. In a study in Spain, interactions between four different species (cattle, domestic pigs, red deer, and wild boar) in a bTB endemic system found that although there was a low percentage of direct interactions between these species, there was a high percentage of indirect interactions over the 3-day time frame investigated, suggesting a high risk of indirect transmission (109). A similar study in France detected a high frequency of indirect interactions between badgers, wild boar, and red deer at waterholes and baited locations (110). Therefore, environmental contamination may present risks for transmission to susceptible hosts sharing the same resources as infected individuals. However, in addition to the presence of an infected host that is shedding, the pathogen must remain viable in the environment for enough time to encounter the susceptible host.

#### Routes of Transmission of *M. bovis* to Rhinoceros in Sub-Saharan Africa

Predicting routes of transmission of MTBC requires an understanding of patterns of shedding, movement patterns, social behavior, and resource utilization of susceptible hosts in relation to infected hosts, and the persistence of the pathogen in a contaminated environment.

##### Wildlife Maintenance Hosts as a Source of *M. bovis* Infection in African Rhinoceros in Sub-Saharan Africa

Domestic livestock (such as cattle) are implicated as bTB maintenance hosts where spillover into wildlife occurs (25, 111). However, since KNP and HiP have perimeter fencing in place to prevent disease interactions between wildlife and cattle, transmission from livestock is unlikely to be a major mode of *M. bovis* infection acquisition in rhinoceros in these areas. In South Africa, the African buffalo is a recognized bTB reservoir host that is implicated in the spill-over of *M. bovis* to other susceptible hosts, both directly, and indirectly through shedding into the environment (27, 68, 105, 112). There is evidence that greater kudu can also be maintenance hosts in the Kruger National Park and possibly in other bTB endemic areas where the species occurs (27). Since these large herbivorous hosts are often found in similar ranges and utilize the same resources as white and black rhinoceros, interactions between these species are likely to occur. These interactions may be a potential route for transmission of *M. bovis* to African rhinoceros.

According to recent biodiversity statistics, KNP African rhinoceros share the park with an estimated 37,130 African buffaloes (21). Similarly, HiP has a buffalo population of ~3,500 (113). African buffaloes are socially organized into herds, which can be as large as 1,000 individuals (112, 114). A study that investigated seasonal movements and habitat use by these animals revealed home ranges varying between 73 and 601 km^2^ (115). Due to the size of their home ranges, interactions with other species (including rhinoceros), particularly at aggregation points such as water sources or shared feeding areas, are likely to occur at a relatively regular frequency. Dispersal events, though less frequent in adult females, occur in adults of both sexes of buffalo (116). Natal dispersal events occur at least once in most adult male buffaloes, and can be driven in both sexes by seasonal (water and nutrient) or social resource limitations (117). Additionally, bTB disease may influence individual health and body condition in buffaloes, which could indirectly impact dispersal events (118). The resulting frequency of dispersal events may influence the probability of pathogen exposure opportunities resulting in spillover from buffaloes to other susceptible species, including white and black rhinoceros in bTB endemic areas.

Investigation of preferred vegetation and habitat of buffaloes showed the strongest association with open to closed herbaceous vegetation on temporarily flooded land, closed shrubs, open shrubs or with 40–65% crown cover (115). The white rhinoceros, like the African buffalo, is a grazing species (119, 120). Their vegetation preference closely mirrors that of buffaloes. In wet months, white rhinoceros may concentrate their grazing in the short grass-dominated grasslands, while in the dry seasons, they move to tall grass grasslands, with a general preference for shaded grasses. Thickets are generally rejected in favor of open grassland vegetation. The black rhinoceros is a browsing species, and their vegetation preference has less in common with that of African buffaloes. They tend to associate closely with thickets (closed shrubland or low forest areas) for access to food (120). For this reason, pathogen exposure interactions with buffaloes due to aggregation at shared feeding areas may be more likely to occur in white rhinoceros than in black rhinoceros.

Available statistics indicate that there are between 11,200 and 17,300 greater kudu in KNP (21). For HiP, a recent estimate of the greater kudu population was not found. The greater kudu is a browsing species of antelope that is socially organized into small bachelor, cow, or mixed herds, typically of fewer than ten individuals (121–123). The home ranges of these herds are typically small and stable, and male home ranges often overlap; the greater kudu social system appears to be based on absolute social dominance (according to age) and territoriality is not evident in this species. Due to their small and stable home ranges, interactions with other species (even indirect) are likely to be less frequent than those observed in buffaloes, who range more widely. However, black rhinoceros share their vegetation preference of thickets or more woody, covered vegetation with this species (124); as a result, indirect interactions with infected kudu (e.g., via shedding of *M. bovis* through fistulated lymph nodes in kudu leading to contamination of vegetation during browsing) may be an important mode of bTB transmission to black rhinoceros. Buffaloes, greater kudus, black and white rhinoceros share water pans, which may also increase the frequency of interactions within and between these species (125–128).

Overall, the wide ranges traversed by buffaloes on a seasonal basis and the potential for contamination of browse by infected kudu, coupled with evidence supporting their integral roles as maintenance hosts for bTB, support the potential for *M. bovis* transmission to white and black rhinoceros (115, 129). While overlapping vegetation preference, mud wallow usage, and ranges may be important infection predictors, indirect transmission of *M. bovis* from maintenance hosts may not be the only risk factor for infection of rhinoceros. Risk factors for infection should be considered as part of a multi-dimensional network, with the potential for transmission from other infected species, or possibly intra-species transmission.

##### Intra-Species Transmission of MTBC in Rhinoceros

Initial *M. bovis* infection in free-ranging rhinoceros in KNP was likely a result of spread from African buffaloes or other infected wildlife species, since the strains of *M. bovis* isolated from rhinoceros cases were the same as those identified in other KNP wildlife, based on comparison of spoligotypes in different studies (19, 64). However, it is unclear whether these infections were the immediate result of inter- or intra-species transmission in rhinoceros. Although rhinoceros have been translocated extensively, it is interesting that the only reported cases of bTB in rhinoceros in South Africa are in animals that originated from or spent time in parks with *M. bovis*-infected reservoir hosts (9, 17, 19). Therefore, further investigation is needed to determine if there is a risk of intra-species spread in rhinoceros.

As with inter-species transmission, the risk of intra-species transmission is dependent on whether the infected host is shedding MTBC, and the frequency of interactions between shedding and susceptible individuals, either directly or indirectly through utilization of shared resources. Evidence suggests that rhinoceros can shed MTBC into their environment in respiratory secretions, or at least that mycobacteria are present in the respiratory system of infected individuals (43, 130). Necropsies of two black rhinoceros in an Indian zoo revealed the presence of acid-fast organisms and large volumes of purulent material in the lungs (14). One of these rhinoceroses was sneezing and had a yellow muco-purulent nasal discharge in the days before its death. *M. tuberculosis* has also been isolated from nasal secretions from an infected black rhinoceros in a zoo which had diagnosed TB in several different species of animals (16). In addition, *M. tuberculosis* was isolated from a gastric lavage sample of a captive black rhinoceros with pulmonary disease, which suggests that like in humans, infected material may be coughed up and swallowed, leading to potential shedding of mycobacteria in feces (8, 15). These observations suggest the possibility that infected rhinoceros may transmit MTBC in secretions, presenting a risk for spread to other animals, and possibly humans, that are in close prolonged contact, such as in a zoo setting.

In free-ranging African rhinoceros, characteristics of social organization may inform the frequency of interactions between shedding and susceptible individuals. Adult female rhinoceros tend to occupy home ranges of up to 70 km^2^, whereas adult bulls are often territorial, and occupy ranges up to 40 km^2^ with little to no overlap, although young bulls may share their territory (131, 132). Young adult females generally range more widely than males, then settle into a similar, smaller home range to have their first calf (131). Home ranges are typically based on permanent water sources and food availability. African rhinoceros may move beyond their usual home ranges during dry periods and peak mating months (124, 133). Although there are some cases in which social groups have been observed in black rhinoceros (134), they tend to be more solitary. In general, cohesive social groups of white rhinoceros are mostly pairs—these can be adolescent-adolescent, cow-adolescent, cow-cow, and cow-calf pairs (132). However, social groups of up to ten individuals have been observed in white rhinoceros in the KNP (134). As density of white rhinoceros in a home range increases, the range occupied by individual cows or territorial bulls decreases. Groups of up to four adult cows with their offspring generally have smaller home ranges than solitary cows (132).

Behavioral characteristics of rhinoceros may also influence the risk of intra-species transmission of pathogens. Territorial behavior is prominent in adult bulls, with frequent olfactory territorial marking, or urine “spraying.” Additionally, both defecation and urination are ritualized in territorial bulls, using specific locations, called “middens,” scattered around the territory (131, 133, 135). Therefore, if *M. bovis* is excreted in feces, like in wild boar and red deer (106), middens might serve as a contaminated site where MTBC bacteria persist. In both black and white rhinoceros, mud wallowing is a behavior practiced more frequently during summer, and in the heat of the day, but can occur at any time (133). Therefore, shared use of wallows may be a potential source of intra-species transmission of *M. bovis* excreted in respiratory secretions or feces.

In summary, both direct and indirect interactions occur between individual rhinoceros, with direct contacts likely occurring at a higher frequency in white rhinoceros. Social exchanges, as well as overlap of ranges, and shared utilization of water sources, middens, and mud wallows are likely to result in indirect interfaces that carry potential for pathogen transmission. However, the apparent low prevalence and lack of disseminated disease in affected free-ranging populations of rhinoceros suggest that the risk of intra-species transmission of MTBC is lower than in captive rhinoceros (19), due to differences in duration and frequency of contact.

##### Environmental Contamination as a Route for Indirect Transmission of *M. bovis*

For indirect transmission of bTB to occur, an area, shared by the recipient host, must be contaminated with MTBC by an infected individual (109). Several studies have successfully isolated pathogenic mycobacterial DNA from various environmental substrates, including water, soil, sediments, and grass; this finding supports the possibility of a *M. bovis*-contaminated environment as a source of exposure for rhinoceros (136). Recipient host exposure risk likely increases with an increase in the mycobacterial load shed by infected hosts into the environment.

Factors affecting persistence of *M. bovis* in the environment have been investigated but are still poorly understood. Because of the low sensitivity associated with culture of MTBC from the environment, qPCR specific for MTBC DNA has been adapted as a supplementary quantification method (136, 137), and 16S rRNA has also been used as a proxy for viable MTBC (138). In addition, the type of samples collected within a system also appears to influence detection of *M. bovis*. For example, using PCR, MTBC DNA has been more frequently recovered in sediments around waterholes than in water in environments with *M. bovis*-infected hosts (139).

Seasonal changes in environmental conditions appear to influence persistence of *M. bovis* in the environment. Both air and soil temperatures affect detection of MTBC DNA in environmental samples (136, 137, 139). Soil concentrations of *M. bovis* DNA were higher in spring compared to all other seasons in the Iberian Peninsula (139). Regardless of soil type, *M. bovis* DNA concentrations were higher when air and soil temperatures were moderate (averaging ~15°C and 17°C, respectively), and with greater soil moisture content (~50%) in spring, compared to higher air and soil temperatures (maximum averages 32.6 and 26.4°C, respectively) and lower soil moisture content (~2%) in summer in this region (139). A study in Michigan (USA) showed that the persistence of *M. bovis* (measured by PCR and culture) in contaminated environmental substrates, which varied between 4 weeks and 6 months, was shortened by exposure to high ambient temperatures, increased intensity of solar radiation, and higher loss of substrate moisture through evapotranspiration (136). In addition, when soil was spiked with *M. bovis*, persistence was longer at 4°C than at 22°C (137). This was in agreement with field studies that showed that *M. bovis* persisted longer in soil in autumn/winter than in summer in Michigan and New Zealand (140, 141), though in these cases it is not clear whether this was only correlated to temperature, or a combination of variables associated with certain seasons. One previous study done under controlled conditions presented contradictory findings related to the correlation of *M. bovis* persistence with temperature; *M. bovis* persisted longer in spiked soils at 37°C than at 4°C (138), highlighting some of the continued knowledge gaps related to persistence of *M. bovis* in the environment. Physico-chemical properties in soil, such as the proportional contribution of clay, silt, sand and organic matter to overall composition, as well as pH, and mineral content (137), may also affect *M. bovis* persistence in the environment. Presence of shade has also been associated with the persistence of environmental *M. bovis*, likely due to maintenance of higher water content and moderate temperatures of the soil and vegetation (142). Additionally, reduced ultraviolet radiation in shade results in less cell stress and fewer genetic mutations, improving bacterial survival (142). These areas may play a role in exposure to *M. bovis* because resting rhinoceros and other species often occupy shady areas during the hottest times of the day, which may promote concentration of bacteria shed in secretions.

In addition to abiotic factors, there are other biological reservoirs that are ubiquitous in the environment and may play a vital role in environmental persistence and subsequent transmission of MTBC. MTBC bacilli have been isolated from free-living amoeba, found frequently in soil (143). It has also been discovered that earthworms (*Lumbricus terrestris*) can disseminate *M. bovis* from contaminated animal feces to the surrounding soil through casting egestion (144). These worms can shed bacteria for up to 4 days after initial ingestion of contaminated feces. The presence of these organisms in areas where grazing by *M. bovis*-infected and susceptible hosts occurs may promote exposure through indirect interactions.

It is hypothesized that, due to the influence of environmental variables on the ability of *M. bovis* to persist in the environment, there may be a seasonal variation in bTB transmission risk. Environmental persistence of *M. bovis* under cold and wet conditions, as well as seasonal changes in the presence of shade and vegetation, may influence *M. bovis* exposure risk in rhinoceros; however further studies are needed to determine pathogen persistence in the natural habitats of these species.

## Discussion

### The Importance of MTBC Infections in African Rhinoceros

Today, the largest free-ranging populations of African rhinoceros in South Africa are located in bTB endemic areas. While poaching of African rhinoceros for their horns is currently the most substantial threat to their conservation (23, 24), the presence of bTB in rhinoceros presents a considerable barrier to conservation due to the inability to translocate animals from bTB endemic areas to safeguarding areas that are bTB free. Without the tools to screen rhinoceros in endemic areas for *M. bovis* infection, regulations prevent translocation to bTB-free areas. Individuals that cannot be moved for safeguarding purposes are then exposed to risk of mortality resulting from poaching incidents. Although bTB is not currently recognized as a major cause of morbidity or mortality or a threat to rhinoceros population health, understanding the epidemiology and pathogenesis of this disease in rhinoceros will provide a foundation for studying the impact of bTB on these species. African rhinoceros populations in zoos around the world may also be at risk of MTBC infection, as individuals in these populations have exhibited morbidity and mortality (4–19). Based on limited case reports, both *M. tuberculosis* and *M. bovis* infect black and white rhinoceros, although the epidemiology and sources of these infections may differ. In addition to the impact on individual rhinoceros health, these infections may also result in spread to other animals, as well as humans, in the zoo environment (12, 13, 130). Therefore, it is essential to investigate the epidemiology of MTBC infections in rhinoceros in various settings to inform the most appropriate disease management and control strategies.

### Hypothesized TB Risk Factors

As in other species, it is expected that the risk of MTBC infection in rhinoceros will be based on factors influencing susceptibility of the individual and exposure to the MTBC (28).

#### *M. tuberculosis* vs. *M. bovis*

Most reported cases of TB in captive rhinoceros resulted from infection with *M. tuberculosis*, with only a few cases caused by *M. bovis*. This is likely due to differing levels of exposure to each pathogen according to its prevalence, as well as the likelihood of interaction with an infected host, including both other animals and humans. TB affects human populations worldwide, and most human cases are caused by infection with *M. tuberculosis* (145). Captive rhinoceros may therefore have a higher likelihood of exposure to *M. tuberculosis* than *M*. *bovis* through their prolonged close contact with infected human caregivers. In these cases, transmission may occur through aerosols or through a contaminated environment, and possibly both.

While *M. bovis* can cause TB in humans, the pathogen is less efficient than *M. tuberculosis* at propagating through human hosts (146). This animal-adapted MTBC species predominantly occurs in livestock and wildlife populations, and is maintained in certain endemic areas by susceptible host populations (3). Free-ranging rhinoceros in bTB endemic areas may be exposed to *M. bovis* through infected hosts or a contaminated environment; the latter is likely to occur more frequently, especially for inter-species transmission, as free-ranging animals are less likely to have close prolonged contact with other infected host species than they are to share aggregation points in their environments (e.g., water sources) with these species. Exposure to *M. tuberculosis* is less likely in free-ranging populations than in captive rhinoceros, as they have little to no interaction with humans, which are the most affected by this pathogen.

#### Species-Specific Susceptibility

The more frequently reported occurrence of TB disease in captive black rhinoceros than in white rhinoceros, from the case series outlined in Figure 2 and Supplementary Table 1, lends credence to the hypothesis that black rhinoceros are more prone to TB disease than white rhinoceros. However, this observation may be due to other confounding factors, including inapparent differences in numbers of black and white rhinoceros in captivity, and differences in *M. tuberculosis* or *M. bovis* exposure. Disease surveillance and reporting biases also exist because not all institutions that house rhinoceros conduct post-mortem TB surveillance or have equal diagnostic capabilities, not all cases of TB in rhinoceros are recorded in the literature, and non-cases are often not reported (147). Nevertheless, the data presented provides foundational hypotheses for further evaluation of TB risk in black and white rhinoceros.

#### Sex as a Risk Factor for MTBC Infection and TB

Limited available data from scientific reports on rhinoceros show more cases of TB in males compared to females (Figure 2 and Supplementary Table 1). It is unknown whether this observation represents a true sex predilection for TB, or whether reports are biased toward males for other reasons. That said, a proposed hypothesis is that African rhinoceros males have a higher susceptibility to TB than females, and this is further supported by males in other species tending to have higher rates of TB compared to females (refer to section Demographic Risk Factors).

In free-ranging African rhinoceros populations in South Africa, it is hypothesized that females have a higher exposure to *M. bovis* due to their wider home range, and more consistent association in cohesive groups than males, and it is unknown how this may contribute to the overall TB risk of males and females in these populations. More controlled investigation in free-ranging populations is required to test these hypotheses.

#### Age as a Risk Factor for MTBC Infection and TB

All recorded TB and bTB cases (Figure 2 and Supplementary Table 1) have occurred in adults. One explanation for this observation is the chronic and recurring nature of this disease. It is possible that young individuals are just as susceptible to MTBC infection as older individuals, but that the disease takes extended time to clinically manifest. An alternative explanation is that diminishing immunocompetence with age (as occurs with age-based hormonal changes in humans as well as the immune effects of old age) could render adults more susceptible to infection and onset of disease than young rhinoceros. It is possible that this observation is attributed not only to age-based changes in susceptibility, but is also due to age-related changes in exposure to either *M. tuberculosis* and *M. bovis* due to translocation of captive rhinoceros for management and breeding (Figure 2 and Supplementary Table 1) (4). Based on this limited information, and age-related TB risk trends observed in most other species, it is hypothesized that susceptibility to MTBC infection and disease progression with exposure increases with age in black and white rhinoceros species.

In free-ranging populations of African rhinoceros in SA, incidence of *M. bovis* is expected to increase with age, due to more intense, and consistent exposure of adults in their smaller, more “settled” home ranges compared to that of the more nomadic young, and the accumulation of repeat exposures over their lifetime. The prevalence of *M. bovis* may also be higher in older animals, reflecting the chronic nature of disease in these long-lived species. The impact of increasing age on the overall TB risk of these populations is still unknown, and requires more investigation.

#### Environmental and Seasonal Factors Affecting Exposure to and Transmission of *M. bovis* in Free-Ranging Rhinoceros in South Africa

The characteristic closer association of black rhinoceros with closed, shady environments than white rhinoceros may increase exposure to *M. bovis* in this species; this is expected to occur as a result of their sharing of this habitat with the suspected bTB maintenance host, the greater kudu, as well as the longer persistence of *M. bovis* in shady (vs. irradiated) conditions. Conversely, the more frequent association of white rhinoceros with soil-associated bTB reservoirs and African buffaloes (a prominent bTB maintenance host) with whom they share their environment and food source, is likely to increase their *M. bovis* exposure. Additionally, because the white rhinoceros is considered a more social species than the black rhinoceros, it is hypothesized that *M. bovis* risk resulting from intra-species interactions will be comparatively higher in white rhinoceros. Overall, because of the higher population of buffaloes (the maintenance host with which white rhinoceros is expected to more frequently associate) than greater kudus (with which the black rhinoceros is expected to more frequently associate), as well as the expected occurrence of more intra-species interactions in white rhinoceros, it is hypothesized that there will be a higher risk *M. bovis* exposure in white rhinoceros populations than in black rhinoceros populations in KNP and HiP.

In free-ranging African rhinoceros populations in South Africa, it is hypothesized that there is an association between seasonal fluctuations in environmental conditions, rhinoceros spatial patterns, and *M. bovis* infection risk. During hot or dry periods, the *M. bovis* exposure of rhinoceros (and other species) may increase due to increased aggregation of *M. bovis-*infected and susceptible hosts at water sources. During hotter periods, specifically, the aggregation of infected and susceptible hosts in shady areas and/or mud wallows may be associated with increased *M. bovis* exposure. Seasonal fluctuations in soil-associated bTB reservoirs such as free -living *M. bovis* in the soil, as well as earthworms and amoeba, may also be associated with seasonal changes in incidence *M. bovis* infection in African rhinoceros and other animals.

### Future Research

The most pressing concern related to TB in African rhinoceros is the acquisition of knowledge and the development of tools to inform surveillance and control strategies for the disease, as well as conservation plans.

The development of sophisticated diagnostic tools may allow for early detection of infection; this would enable earlier interventions that could improve the prognosis of infected individuals and mitigate the spread of the infection in captive and free-ranging systems. Of particular consideration is logistical feasibility and fitness-for-purpose of a test. Capture and immobilization of rhinoceros, especially in free-ranging populations, is extremely costly and is a source of stress for the animal. Therefore, the use of a test like the TST, in addition to being unreliable in this species, would be ill-advised, as it involves immobilization for both administration and interpretation of the test on separate occasions, increasing the cost and the stress for the rhinoceros undergoing testing. Development of blood-based cytokine release assays for bTB in African rhinoceros is currently ongoing; these tests require a single capture and immobilization, and once validated, may be reliable, cost effective diagnostic methods for bTB in rhinoceros.

Coordinated studies in captive populations may help to clarify demographic factors (e.g., age, sex, species) as risks for *M. bovis* infection and disease progression in African rhinoceros. This would involve ante-mortem monitoring for MTBC infection, as well as thorough post-mortem exams that include histopathology and ancillary diagnostic tests. Because zoological facilities tend to keep curated medical records, retrospective, longitudinal data may already be available to address these knowledge gaps. Careful, standardized data curation across institutions could inform and enumerate a study population and be used to identify cases and non-cases. In any such study, attention to confounding factors such as differences in exposure to MTBC based on animal origin, movement history, and TB prevalence in human populations should be considered.

In South Africa, population-based epidemiological studies of bTB in free-ranging African rhinoceros populations are currently ongoing. Findings from such studies could help identify major drivers of bTB infection in free-ranging populations as well as identify low risk individuals, which would have immediate benefit to current conservation and translocation efforts. This knowledge could be applied to inform management decisions for these populations, e.g., to minimize the probability of moving a false-negative infected animal out of bTB endemic areas and inadvertently spreading bTB to other areas. Such studies may also aid in identifying important bTB risk mitigation opportunities aimed to decrease continued spread of bTB in black and white rhinoceros living in these fragile ecosystems.

## Conclusion

This review has focused on available literature that could help to characterize the risk posed by MTBC (including *M. bovis* and *M. tuberculosis*) to African rhinoceros species. It has also drawn attention to major knowledge gaps pertaining to TB in rhinoceros. By identifying and systematically addressing each of these knowledge gaps, advances will inform management decisions for conservation of African rhinoceros, and South African biodiversity.

## Author Contributions

RD, MM, WG, and CW reviewed available literature and wrote and edited the manuscript. PB edited the manuscript. RD and MM generated Figures 1, 2. All authors read and approved the final manuscript.

## Conflict of Interest

The authors declare that the research was conducted in the absence of any commercial or financial relationships that could be construed as a potential conflict of interest. The reviewer BB declared a past co-authorship with one of the authors PB to the handling editor.
