# Supplementary material for: Epidemiology of Tuberculosis in Multi-Host Wildlife Systems: Implications for Black (Diceros bicornis) and White (Ceratotherium simum) Rhinoceros
Source: Front Vet Sci. 2020 Nov 4;7:580476. doi: 10.3389/fvets.2020.580476 (PMC7672123; doi:10.3389/fvets.2020.580476)
Supplement: Supplementary file 1 [file Table_1.DOCX]

**Supplementary Table 1:** Globally recorded historical cases of tuberculosis in black rhinoceros (*Diceros bicornis*) and white rhinoceros (*Ceratotherium simum*) (4–19)

| Year of TB diagnosis | Location | Living conditions (captive/ semi-captive/free-ranging) | Additional notes on rhinoceros movements | Species | Age (years) at time of death | Sex | MTBC Species | Key Finding | Place of origin * | Ref (s) |
| --- | --- | --- | --- | --- | --- | --- | --- | --- | --- | --- |
| 1892 | Wroclaw,  Poland | Captive (zoo) | ... 🡪 Travelled with Hagenbeck’s Nubian African show for 10 years 🡪 Wroclaw, (1888). | Black rhinoceros | ≥14 | Unknown | Unknown | Died of TB | From wild | (4) |
| 1944 | Dresden, Germany | Captive (zoo) | ... 🡪 Dresden (1928) | Black rhinoceros | ≥16 | Male | Unknown | Died of TB | From wild  East Africa | (4) |
| 1956 | Pretoria, South Africa | Captive (zoo) | Mkhuze 🡪 NZG (1944) | Black rhinoceros | 12-13 | Male | Unknown | Died of TB | From wild – Mkhuze, South Africa | (4,5) |
| 1957 | Cologne, Germany | Captive (zoo) | ... 🡪 Cologne (1953) | Black rhinoceros | ≥4 | Male | Unknown | Died of TB | From wild | (4) |
| 1961 | Osaka  Japan | Captive (zoo) | ... 🡪 Osaka (1955) | Black rhinoceros | ≥6 | Male | Unknown | Died of TB | From wild | (4) |
| 1969 | Prague, Czech Republic | Captive (zoo) | ... 🡪Imported by Behrend (dealer?) 🡪 Prague (1954) | Black rhinoceros | ≥15 | Male | Unknown | Died of TB | From wild | (4) |
| 1970 | Hluhluwe-iMfolozi, South Africa | Free-ranging | No record of any translocation | Black rhinoceros | Old; age not specified | Female | Unknown | Condition deteriorated up to death, general poor health. Acid fast *Mycobacterium* sp. isolated from lung granulomas at necropsy. | From wild (HiP) | (6) |
| 1978 | USA  Washington DC | Captive (zoo) | ... 🡪 purchased from “Dealer A” Washington D.C. (1961) | Black rhinoceros | 20 | Female | *M. bovis* | Ante-mortem diagnosis- died of TB after 3 day- illness during a time period of extreme heat & humidity. | From wild  East Africa | (4,7) |
| 1979 | USA  Washington DC | Captive (zoo) | ... 🡪 purchased from “Dealer A” 🡪 Washington D.C. (1960) | Black rhinoceros | Unknown | Male | *M. bovis* | Ante-mortem diagnosis – *M. bovis* isolated from lung biopsy. Euthanized. | From wild  East Africa | (4,7) |
| 1984 | San Diego  USA | Captive (zoo) | Hannover, Germany (1968) 🡪 Budapest (1969) 🡪 San Diego Wild Animal Park (1983) | Black rhinoceros | 16 | Female | *M. tuberculosis* | Died of TB | Captive born -Hannover, Germany | (4,9,10) |
| 1990 | Hluhluwe iMfolozi, South Africa | Free- ranging |  | Black rhinoceros | Unknown | Unknown | Unknown | Necropsy reveals lesions consistent with TB; no culture confirmation | From wild (HiP) | (11) |
| 1990 | USA | Captive (zoo) |  | Black rhinoceros | Unknown | Unknown | *M. tuberculosis* | - | - | (9) |
| 1991 | Audubon,  New Orleans,  USA | Captive (zoo) | Umfolozi, South Africa 🡪 NY Bronx (1962) 🡪 New Orleans (1974) | White rhinoceros | 29 | Male | *M. bovis* | Died of TB and pneumonia | From wild (HiP) | (4,12,13) |
| 1992 | Mysore, India | Captive (zoo) | No translocation.  Parents were purchased by the zoo from the same dealer, were captured as adults from wild. | Black rhinoceros | 13 | Female | *M. tuberculosis* | Died of TB | Captive born - Mysore, India | (4,14) |
| 1993 | Chicago, USA | Captive (zoo) |  | Black rhinoceros | 31 | Female | *M. tuberculosis* | Positive *M. tuberculosis* cultures from sputum and gastric lavage. Treated – RIF^[[1]](#footnote-1)^, INH^[[2]](#footnote-2)^, PZA^[[3]](#footnote-3)^.  Died 1994: *M. tuberculosis* cultured from thoracic lymph node, tracheal exudates, and gastric lavage. | Origin unknown | (8,15) |
| 1994 | Mysore, India | Captive (zoo) | No translocation. Born to same parents as the rhinoceros in which the previous reported TB case at this zoo (1992) occurred. | Black rhinoceros | 22 | Male | *M. tuberculosis* | Died of TB | Captive born Mysore, India | (4,14) |
| 1998 | Los Angeles, USA | Captive (zoo) | Housed in same facility as previous known TB cases | Black rhinoceros | 24 | Female | *M. tuberculosis* | Nasal wash in 1998 was positive for *M. tuberculosis*, treated with RIF^[[4]](#footnote-4)^, INH^[[5]](#footnote-5)^, PZA^[[6]](#footnote-6)^. Observed weight loss and nasal discharge in 2000, no improvement so it was euthanized in 2001. | Origin unknown | (15,16) |
| 2007 | Limpopo, South Africa | Captive (zoo) | Mkhuze (free-ranging)🡪HiP🡪 Limpopo NZG’s Mokopane Biodiversity Conservation Centre (displaced in Mkhuze by another bull) (2002) | Black rhinoceros | Old (Estimated 35-40) | Male | *M. bovis* | Animal euthanized due to poor condition, old age, dental attrition. Two lung granulomas culture positive for *M. bovis*. | From wild 🡪 Mkhuze | (17) |
| 2013 | Texas, USA | Captive (zoo) |  | Black rhinoceros | 33 | Male | *M. tuberculosis* | Died after non-specific illness. *M. tuberculosis* isolated from lungs at necropsy, spoligotype uncommon in animals but present in humans worldwide. |  | (4,18) |
| 2014 | Private Reserve, South Africa | Free-ranging | - | Black rhinoceros | Adult | Male | *M. bovis* | Dies of pneumonia  *M. bovis* culture positive. | From wild-private reserve, South Africa | (19) |
| 2016 | Kruger National Park, South Africa | Free-ranging | - | Black rhinoceros | Adult | Female | *M. bovis* | Animal euthanized due to poor prognosis. Lung tissue was culture-and PCR- positive/ confirmed *M. bovis*. |  | (19) |
|  |  |  |  | White rhinoceros | Subadult | Male | *M. bovis* | Poor condition of animals attributed drought or poaching wounds – animals were euthanized.  bTB was not considered a cause of poor condition, and no generalized disease or infection in these animals.  *M. bovis* culture positive from one or more of lymph nodes, and/or the lungs. Strain type: SB02121, the most common strain in the KNP. |  | (19) |
|  |  |  |  |  | Adult | Female |  |  |  |  |
|  |  |  |  |  | Adult | Male |  |  |  |  |
|  |  |  |  |  | Subadult | Female |  |  |  |  |
| 2017 | Kruger National Park,  South Africa | Free-ranging | - | White rhinoceros | Subadult | Female |  |  |  |  |
|  |  |  |  |  | Adult | Male |  |  |  |  |

**^*^**Where a rhinoceros originated “from wild” and no additional information is provided, the individual originated from an unrecorded location on the African continent.

This data was located in the Rhinoceros Resource Centre (RRC) literature database, or through extensive web searches with (9) as a guide

1. Rifampicin [↑](#footnote-ref-1)
2. Isoniazid [↑](#footnote-ref-2)
3. Pyrazinamide [↑](#footnote-ref-3)
4. Rifampicin [↑](#footnote-ref-4)
5. Isoniazid [↑](#footnote-ref-5)
6. Pyrazinamide [↑](#footnote-ref-6)
